# Danggui Buxue Extract-Loaded Liposomes in Thermosensitive Gel Enhance* In Vivo* Dermal Wound Healing via Activation of the VEGF/PI3K/Akt and TGF-*β*/Smads Signaling Pathway

**DOI:** 10.1155/2017/8407249

**Published:** 2017-10-24

**Authors:** Meng-Di Cui, Zi-Hao Pan, Li-Qun Pan

**Affiliations:** ^1^First College of Clinical Medicine, Nanjing University of Chinese Medicine, No. 138, Xianlin Avenue, Nanjing 210023, China; ^2^College of Pharmacy, Nanjing University of Chinese Medicine, No. 138, Xianlin Avenue, Nanjing 210023, China

## Abstract

Danggui Buxue extract-loaded liposomes in thermosensitive gel (DBLTG) are a sustained-release local drug delivery system derived from Danggui Buxue decoction, a well-known Chinese herb formula with wound healing potential. In the present study, we investigated the therapeutic effects of DBLTG on dorsal full-thickness excisional wounds in rats by measuring the percentage of wound contraction and hydroxyproline content, as well as conducting histological observations and immunohistochemical analysis. We also assessed involvement of the vascular endothelial growth factor (VEGF)/phosphatidylinositol 3-kinase (PI3K)/Akt and transforming growth factor beta (TGF-*β*)/Smads signaling pathways in the wound healing process upon DBLTG treatment via western blot. The results show that DBLTG treatment remarkably accelerates wound closure, enhances hydroxyproline content in wound granulation tissue, promotes cutaneous wound healing by reducing the inflammatory response and improving fresh granulation tissue formation, and significantly increases the density of blood vessels, cells proliferation, and expression of type I and type III collagen. Moreover, DBLTG markedly upregulates the relative protein expression of VEGFA and TGF-*β*1 and notably stimulates the phosphorylation of Akt and Smad2/3. In conclusion, DBLTG significantly improved dermal wound healing in rats by stimulating angiogenesis and collagen synthesis; these effects are likely mediated via the VEGF/PI3K/Akt and TGF-*β*/Smads signaling pathways, respectively.

## 1. Introduction

Dermal wounds remain a major health problem worldwide. In recent years, wound dressings consisting of biocompatible and biodegradable materials have been developed to improve the wound healing process [1, 2]. Among various wound dressings, thermosensitive gels are particularly suitable as local drug delivery systems for dermal wound healing [3]. Thermosensitive gels provide an ideal semiobliterative moist environment for wound healing [4, 5], which is very important for cell proliferation and collagen synthesis. Moreover, thermosensitive gels are free-flowing at room temperature and, when injected into the wound bed, quickly transform into semisolid gels that fill the entire wound area, protecting the wound and absorbing wound secretions [6, 7]. Additionally, because of the porous structure, thermosensitive gels gradually deliver encapsulated drug into the dermal wound.

Liposomes used as drug carriers for dermal wound healing have many advantages, including desirable biocompatibility, low toxicity, sustained release, increased permeability, and the ability to codeliver hydrophilic and hydrophobic drugs. However, liposomes are relatively unstable, with low viscosity; therefore, there is a need to develop an in situ matrix for drug-loaded liposomes to enhance their stability and increase contact time of the encapsulated drug in cutaneous wounds. One strategy for overcoming these drawbacks includes dispersal of the liposomes into a hydrogel, which is currently used as a platform for ophthalmic, intravesical, and intravaginal drug delivery [8–10].

Danggui Buxue decoction (DBD) is a well-known traditional Chinese medicine (TCM) prescription; it was first described by Li Dong-yuan (Jin and Yuan Dynasty) in his treatise “Nei Wai Shang Bian Huo Lun.” DBD is composed of Danggui (*Angelica sinensis*, AS) and Huangqi (*Astragali radix*, AR) at a ratio of 1 : 5 (g/g) and is known for “invigorating Qi and promoting Xue,” suggesting that it has potential for wound healing. In addition, DBD has been proven to promote proliferation of human umbilical vein endothelial cells (HUVECs) [11]. Recent data indicate that drug serum containing DBD improves the proliferation, migration, and adhesion of endothelial progenitor cells (EPCs), which play a vital role in vascular endothelial regeneration and repair after injury [12]. Our previous studies suggest that ferulic acid coupled with astragaloside IV, considered to be the main active ingredients in DBD, may contribute to angiogenesis in wound healing [13]. However, we did not find any literature regarding DBD as a wound repair agent for local application in dermal wounds. Therefore, this study aimed to evaluate the effects of topical DBD application for promoting wound healing* in vivo*.

Danggui Buxue extract (DBE) derived from DBD was loaded into liposomes in thermosensitive gel for topical administration. Subsequently, we investigated the therapeutic effects of DBE-loaded liposomes in thermosensitive gel (DBLTG) on dorsal full-thickness excisional wounds in rats and assessed involvement of the vascular endothelial growth factor (VEGF)/phosphatidylinositol 3-kinase (PI3K)/Akt and transforming growth factor beta (TGF-*β*)/Smads signaling pathways in dermal wound healing processes after DBLTG treatment.

## 2. Materials and Methods

### 2.1. Chemicals and Reagents

AS and AR were purchased from Fengyuan Tongling Prepared Chinese Herb Co., Ltd. (Anhui, China) and authenticated by Professor Xun-Hong Liu (Department of Science of TCM Identification, College of Pharmacy, Nanjing University of Chinese Medicine). Soybean phospholipid and cholesterol were provided by A.V.T. Pharmaceutical Co., Ltd. (Shanghai, China). Ferulic acid and calycosin-7-O-*β*-d-glucoside were purchased from National Institute for Food and Drug Control (Nanjing, China). Poloxamer 407 (P407), Poloxamer 188 (P188), and hydroxypropyl methyl cellulose (HPMC) were purchased from BASF (Ludwigshafen, Germany). The other reagents were of analytical grade.

### 2.2. Preparation and Characterization of DBLTG

AS (6 g) and AR (30 g) were sliced into small pieces, mixed, and then boiled twice in distilled water at a ratio of 1 : 15* (w/v)* with refluxing for 45 min each time. The herb residue was removed by filtration, and the filtrate mixture was concentrated into 72 mL via a rotary vacuum evaporator (Heidolph, Germany). The concentrated filtrate (0.5 g crude herb/mL) was purified by alcohol precipitation to prepare DBE at the same concentration. Liposomes were prepared via a thin-film dispersion-ultrasonic method as described by Bochot et al. [14]. Specifically, lipid materials, soybean phosphatidylcholine, and cholesterol (100 mg : 16.6 mg) were dissolved in 2.5 mL absolute ethanol, evaporated to dryness in a 100 mL round-bottomed flask at 40°C using a rotary vacuum evaporator, and desiccated overnight at 37°C under vacuum. To prepare DBE-loaded liposome suspensions, the dried lipid film was rehydrated with 4 mL DBE at a concentration of 0.5 g crude herb/mL in 100 mL round-bottomed flasks using a rotary evaporator at 40°C and 100 r/min for 1 h. To prepare blank liposome suspensions, the dried lipid film was rehydrated with 4 mL Milli-Q water (Millipore, USA) under the same conditions. Subsequently, the two suspensions were sonicated by an ultrasonic probe at 400 W for 5 min and then extruded thrice through a 0.2 *μ*m polycarbonate membrane (Whatman, GE Healthcare, UK) with an extruder (Avestin, Canada) to reduce the size of the liposomes. DBE-loaded liposomes (DBLs) were separated from the free components in DBE via a macroporous resin absorption column (2.0 cm × 15.0 cm) presaturated with blank liposomes (BLs). Finally, sterile thermosensitive gel ingredients (25%* w/v* P407, 2.5%* w/v* P188, and 0.3%* w/v* HPMC) were dispersed into DBLs or BLs by means of constant magnetic stirring at 4°C and 100 r/min, resulting in synthesis of DBLTG or blank liposomes in thermosensitive gel (BLTG).

The particle size and polydispersity index (PDI) of the liposomes were measured via a Zetasizer Nano ZS90 (Malvern, UK). The encapsulation efficiencies (EE) of AS and AR in DBLs were determined by quantifying ferulic acid and calycosin-7-O-*β*-d-glucoside identified as the index component of AS and AR, respectively, per the Chinese Pharmacopoeia (2010) using a high-performance liquid chromatography with diode array detection (HPLC-DAD) method [15]. The stability parameters of DBLs including particle size, PDI, and leakage rate (LR) were evaluated at 4°C during the first four weeks after liposomes preparation. The sol-gel transition temperature and gelation time of DBLTG were measured by a tube-inverting method with a 5 mL vial immersed in a thermostatic water bath at an increment of 0.5°C per step. EE and LR was calculated according to (1) and (2), respectively, as listed below:

(1) EE=Experimental  drug  loadingTheoretical  drug  loading×100%,

(2) LR=EE0−EEtEE0×100%,

where EE_0_ was the original encapsulation efficiency of AS or AR and EE_*t*_ was the encapsulation efficiency of AS or AR at week *t* after liposomes preparation.

### 2.3. Animals

Fifty-four adult male Sprague-Dawley (SD) rats, weighing 180–220 g, were provided by Slac Laboratory Animal Ltd. (Shanghai, China). The rats were raised under controlled conditions, including 22 ± 2°C, 50–60% humidity, 12 h light-dark cycle, and free access to food and water. All the animal care and experimental protocols were evaluated and approved by the Animal Investigation Ethics Committee of Nanjing University of Chinese Medicine.

### 2.4. In Vivo Wound Healing

The animals were randomly divided into the following three groups with eighteen rats each: model control group, vehicle control group, and DBLTG-treated group. Each group was again divided into three subgroups, with six rats each according to sampling time as follows: day 3 group, day 7 group, and day 14 group.

The animals were acclimated in the laboratory for at least one week and fasted overnight with free access to water before surgery. All rats were weighed and then anesthetized with 10% chloral hydrate (3.5 mL/kg* i.p.*). After being shaved with an electric razor to remove all the back hair, the anesthetized rats were disinfected with 2.5% povidone iodine. Two pieces of dorsal skin (1.5 cm × 1.5 cm) were excised from each rat on both sides of the back midline no less than 1.0 cm from the adjacent wound. The skin wound lost the epidermis, most of the dermis, the fat, and the* panniculus carnosus*—exposing the fascia layer.

After surgery, each skin wound was topically given 0.5 mL aseptic BLTG (vehicle control group) or 0.5 mL aseptic DBLTG containing 9.56 ± 0.57 mg AS and 17.52 ± 2.33 mg AR (DBLTG-treated group). Subsequently, each rat was given a surgical dressing and kept in a single cage. At the end of day 3, day 7, and day 14 after injury, images of wound sites were taken, the rats corresponding to the appropriate subgroup were euthanized, and the wound granulation tissue was excised from each animal. A portion of harvested tissue was immediately stored in liquid nitrogen, and the other portion was maintained in 10% buffered formalin for the following tests.

### 2.5. Wound Contraction Assay

Each wound was photographed and measured at each time point; the percentage of wound contraction was then calculated via the following equation [16]: % wound contraction = (initial wound area − present wound area)/(initial wound area) × 100.

### 2.6. Hydroxyproline Content Assay

Hydroxyproline content in wound granulation tissue was examined using a hydroxyproline assay kit from Nanjing Jiancheng Bioengineering Institute (Nanjing, China). This method is similar to that described by Neuman and Logan [17], with slight modification.

### 2.7. Histology

Wound granulation tissue specimens collected at different sampling times were fixed with 10% neutral formaldehyde, dehydrated in gradient alcohol (70% to 100%), embedded in paraffin, and then serially sectioned using a microtome (5 *μ*m). The sections were subsequently deparaffinized and stained with hematoxylin and eosin (H&E) according to a standard protocol. The slices were observed with a microscope to study the morphology of wound granulation tissue according to standard protocols.

### 2.8. Immunohistochemical Staining

Paraffin-embedded sections were deparaffinized and rehydrated, and after antigen retrieval using 10 mM sodium citrate (pH 6.0) for 10 minutes, sections were incubated with primary antibodies against CD34 (Abcam, UK), Ki67 (Abcam, UK), Col1*α*1 (Boster, China), and Col3*α*1 (Boster, China) overnight at 4°C to evaluate neovascularization and cell proliferation, as well as type I collagen and type III collagen synthesis in wound granulation tissue following the manufacturer's protocol.

### 2.9. Western Blot

Briefly, total proteins were extracted from frozen wound granulation tissue specimens with radioimmunoprecipitation assay (RIPA) lysis buffer supplemented with protease inhibitors (Beyotime Biotechnology, China). Lysates were centrifuged at 13220 ×g for 20 min at 4°C, and supernatants were collected. After protein concentrations were determined via the Coomassie brilliant blue protein assay, equal amounts of protein (30 *μ*g) extracted from each sample were resolved by sodium dodecyl sulfate polyacrylamide gel electrophoresis (SDS-PAGE) and transferred onto a polyvinylidene difluoride (PVDF) membrane (0.22 *μ*m, Millipore, USA). The PVDF membranes were blocked with 5% nonfat dry milk (Biosharp, China) in Tris-buffered saline with 1% Tween 20 (TBST) for 1 h at room temperature. After incubating with primary antibodies, including anti-VEGFA (Abcam, UK), anti-PI3K (Cell Signaling Technology, USA), anti-Akt (Cell Signaling Technology, USA), anti-p-Akt (Ser473) (Cell Signaling Technology, USA), anti-TGF-*β*1 (Cell Signaling Technology, USA), anti-Smad2/3 (Cell Signaling Technology, USA), anti-Smad4 (Abcam, UK), and anti-p-Smad2(Ser465/467)/Smad3(Ser423/425) (Cell Signaling Technology, USA) overnight at 4°C, the membranes were washed with TBST at least thrice for 30 min and then incubated with horseradish peroxidase- (HRP-) conjugated secondary antibodies (Bioworld, USA) for 1 h at 37°C. The immunoreactive bands were visualized with enhanced chemiluminescence (ECL) reagent (Beyotime Biotechnology, China) and quantified using Gel-Pro Analyzer software (Media Cybernetics, USA).

### 2.10. Statistical Analysis

All data are expressed as means ± standard deviation. Statistical analysis of data was performed by SPSS Statistics 23.0 (IBM, USA), and the significance of differences among groups was assessed using univariate two-way analysis of variance (ANOVA) followed by Bonferroni pairwise comparisons.* P* values less than 0.05 were considered significantly different from model and vehicle control groups.

## 3. Results

### 3.1. DBLTG Characteristics

The original DBL particle size was 175.53 ± 0.60 nm, with a PDI of 0.28 ± 0.01. The original encapsulation efficiencies (EE_0_) of AS and AR in DBLs were 22.94 ± 1.37% and 8.41 ± 1.12%, respectively. After being stored at 4°C for 1, 2, and 4 weeks, the DBL particle size increased to 184.40 ± 1.08, 191.43 ± 1.55, and 201.53 ± 1.50 nm, with a PDI of 0.29 ± 0.00, 0.30 ± 0.01, and 0.32 ± 0.00, respectively. Moreover, the leakage rate (LR) of AS and AR in DBLs was 14.16 ± 2.02% and 10.97 ± 2.16%, 22.29 ± 2.29% and 24.37 ± 3.53%, and 24.39 ± 4.71% and 28.63 ± 2.87% following storage at 4°C for 1, 2, and 4 weeks, respectively. The results demonstrated that DBLs seemed to be uniform in particle size and maintained quite a stable state at 4°C during the first four weeks after their preparation. DBLTG was a free-flowing gel at room temperature but rapidly transforms into a solid-like gel at 33.00 ± 0.63°C without a crosslinking agent; its gelation time was 2.19 ± 0.18 min at the sol-gel transition temperature. All results were the mean of three independent trials (*n* = 3).

### 3.2. Percentage of Wound Contraction

**Figure 1:**
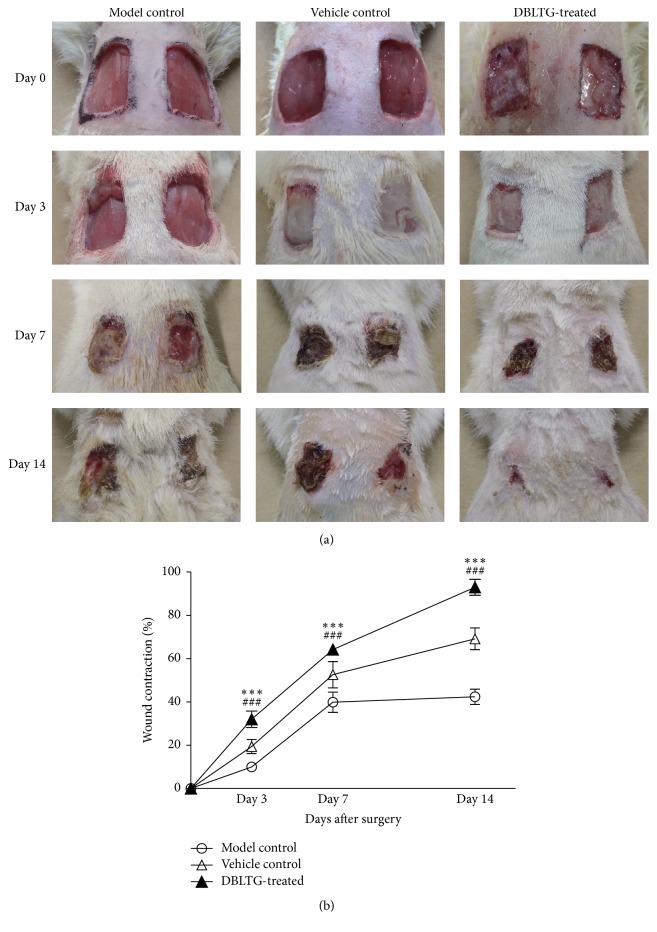
Wound healing process in rats. (a) Macroscopic appearance of dorsal full-thickness excisional wounds in rats from different groups over time after surgery. (b) Percentage of wound contraction in different groups over time after surgery (*n* = 6). ^*∗∗∗*^*P* < 0.001 versus model control; ^###^*P* < 0.001 versus vehicle control. DBLTG, Danggui Buxue extract-loaded liposomes in thermosensitive gel.

Figure 1(a) shows changes in the macroscopic appearance of rat dorsal full-thickness excisional wounds after surgery. Wounds closed faster in the DBLTG-treated group on day 7 and day 14 after surgery, particularly up to day 14, than those in the model control and vehicle control groups. Further, there were no notable differences in the vehicle control and model control groups during the entire wound healing process. As shown in Figure 1(b), the percentage of wound contraction in the DBLTG-treated group was 32.03 ± 3.81, 64.22 ± 2.31, and 92.98 ± 3.64 on day 3, day 7, and day 14 after surgery, respectively; these values were markedly higher than those of the model control and vehicle control groups on the corresponding days (*n* = 6). These results indicate that DBLTG topical administration accelerates cutaneous wound contraction in a rat excisional wound model.

### 3.3. Hydroxyproline Content

**Figure 2:**
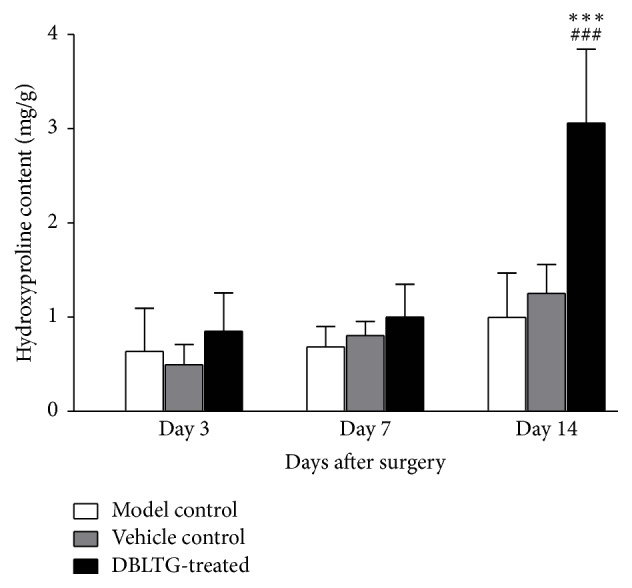
Effect of Danggui Buxue extract- (DBE-) loaded liposomes in thermosensitive gel (DBLTG) on hydroxyproline content in rat wound granulation tissues (*n* = 6). ^*∗∗∗*^*P* < 0.001 versus model control; ^###^*P* < 0.001 versus vehicle control.

Hydroxyproline content in rat wound granulation tissue from the DBLTG-treated group was higher than that in both control groups at each time point after surgery; however, it was remarkably (*P* < 0.001) greater than that in both control groups on day 14 (Figure 2). Moreover, there were no statistical differences between the model control and vehicle control groups during the entire observation process after surgery (*n* = 6). Therefore, differences in hydroxyproline levels in rat wound granulation tissue, especially on day 14 after surgery, are likely attributable to DBLTG administration.

### 3.4. Histology

**Figure 3:**
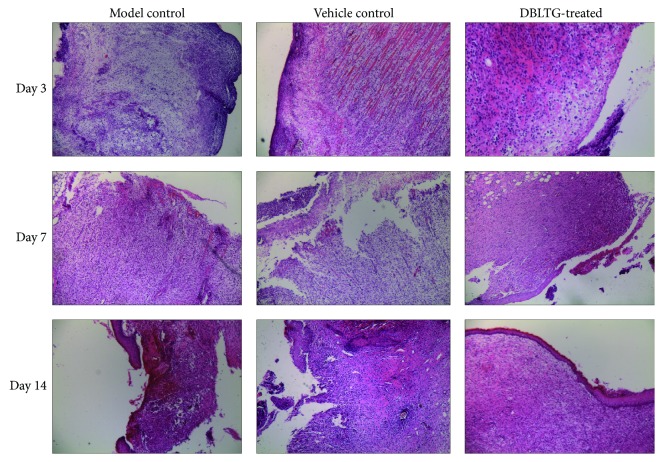
Histological evaluation of wound granulation tissue collected on day 3, day 7, and day 14 after surgery in the three treatment groups. DBLTG, Danggui Buxue extract-loaded liposomes in thermosensitive gel.

To study the tissue reaction to DBLTG, we observed the histology of wound granulation tissue specimens collected on day 3, day 7, and day 14 after surgery and then stained with H&E. As shown in Figure 3, the DBLTG-treated group demonstrated remarkably fewer inflammatory cells and more macrophages and fibroblasts on day 3, while the model control and vehicle control groups showed a greater inflammatory response, along with serious edema and excessive neutrophil infiltration. On day 7, tissue edema had almost disappeared and microvessels and fibroblasts were rapidly growing in an ordered distribution in the DBLTG-treated group. In contrast, tissue edema and inflammatory infiltration were still observed in the vehicle control and model control groups, accompanied by less neovascularization, suggesting that wound healing in both control groups remained in the early stages. On day 14, directional migration of a variety of monocytes, neutrophils, and macrophages induced by inflammatory mediators had occurred; newly formed tissues and a large amount of collagen were homogeneously distributed throughout the entire scaffold; and the regenerative epithelium was almost completely covered with the excisional wounds in the DBLTG-treated group. In contrast, small blank areas with little tissue infiltration remained in both control groups. These histological results indicate that DBLTG accelerates full-thickness excisional wound healing with significant improvements in inflammatory cell infiltration, macrophage and fibroblast proliferation, angiogenesis, collagen synthesis, and reepithelialization, forming a dense and uniform neotissue structure.

### 3.5. Immunohistochemical Staining

To study the effects of DBLTG on angiogenesis, cell proliferation, and collagen synthesis during wound repair, wound tissue samples harvested on day 3, day 7, and day 14 after surgery were immunohistochemically stained with CD34, Ki67, Col1*α*1, and Col3*α*1. Blood vessel density is expressed as integrated optical density (IOD) per unit area of CD34 staining, quantification of proliferating cells was displayed as IOD per unit area of Ki67 staining, and the unit quantity of type I and type III collagen is exhibited as IOD per unit area of Col1*α*1 and Col3*α*1 staining, respectively.

**Figure 4:**
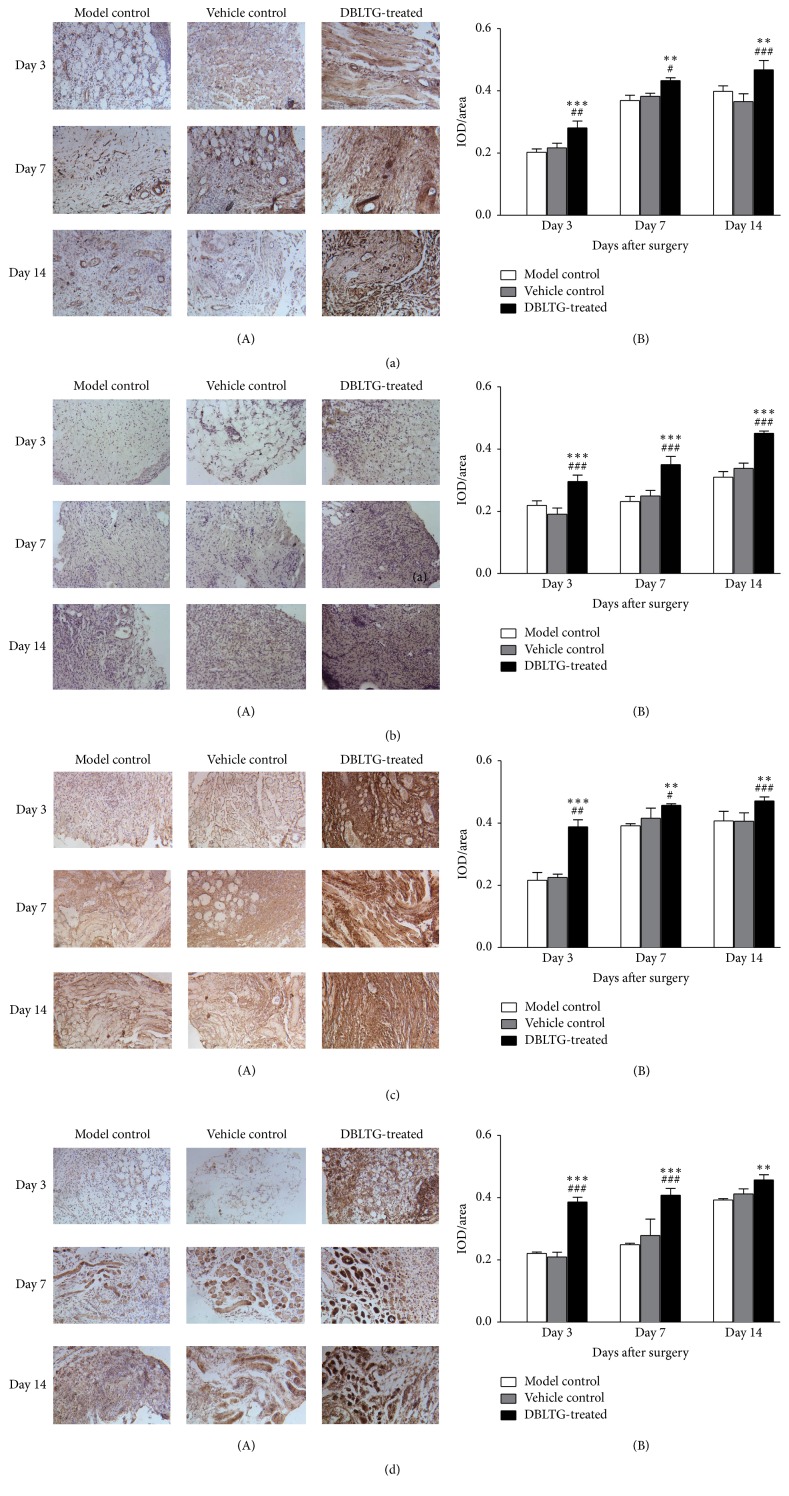
Immunohistochemical staining for CD34 (a), Ki67 (b), Col1*α*1 (c), and Col3*α*1 (d) in rat wound granulation tissue at each time point following surgery in the three treatment groups (*n* = 3). (A) Immunohistochemical images and (B) immunohistochemical analysis results are shown. ^*∗∗*^*P* < 0.01 versus model control; ^*∗∗∗*^*P* < 0.001 versus model control; ^#^*P* < 0.05 versus vehicle control; ^##^*P* < 0.01 versus vehicle control; ^###^*P* < 0.001 versus vehicle control. IOD, integrated optical density; DBLTG, Danggui Buxue extract- (DBE-) loaded liposomes in thermosensitive gel.

CD34 expression in granulation tissues from back full-thickness excisional wounds in rats at each time point after surgery was greater in the DBLTG-treated group than in the model control and vehicle control groups (Figure 4(a)). The DBLTG-treated wounds displayed a notable increase in Ki-67 immunoreactivity as compared with both control wounds, whereas vehicle control wounds exhibited no significant difference in cell proliferative rate compared with model control wounds (Figure 4(b)). The results indicate that DBLTG transplantation can significantly upregulate neovascularization and cell proliferation in wound granulation tissues. Furthermore, Col1*α*1- and Col3*α*1-positive areas in the granulation tissues were remarkably larger in the DBLTG-treated group during the wound healing process than those in either of the control groups (Figures 4(c) and 4(d)). Quantitative analysis demonstrated that type I and type III collagen synthesis were effectively accelerated by DBLTG in accord with increasing hydroxyproline levels. All results were the mean of three independent trials (*n* = 3).

### 3.6. Western Blot Analysis

To investigate molecular mechanisms underlying the therapeutic efficacy of DBLTG on dorsal full-thickness excisional wounds in rats, we evaluated involvement of the VEGF/PI3K/Akt and TGF-*β*/Smads signaling pathways by western blot.

**Figure 5:**
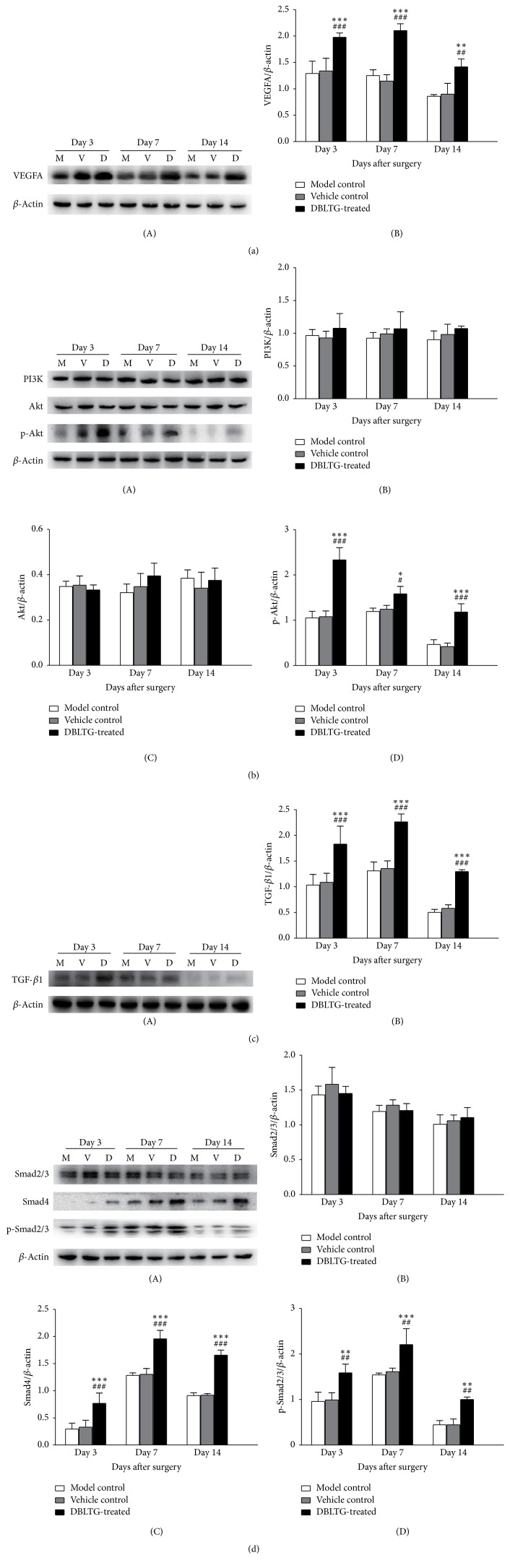
Western blot analysis of vascular endothelial growth factor A (VEGFA) (a); phosphoinositide 3-kinase (PI3K), Akt, and p-Akt (b); transforming growth factor beta 1 (TGF-*β*1) (c); and Smad2/3, Smad4, and p-Smad2/3 (d) expression in wound granulation tissue from different treatment groups at each time point after injury (*n* = 3). (A) Western blot bands and (B) or (B–D) western blot analysis results are shown. ^*∗*^*P* < 0.05 versus model control; ^*∗∗*^*P* < 0.01 versus model control; ^*∗∗∗*^*P* < 0.001 versus model control; ^#^*P* < 0.05 versus vehicle control; ^##^*P* < 0.01 versus vehicle control; ^###^*P* < 0.001 versus vehicle control. DBLTG, Danggui Buxue extract- (DBE-) loaded liposomes in thermosensitive gel; M, model control; V, vehicle control; D, DBLTG-treated.

Angiogenesis is a crucial part of the wound healing process, and it relies on growth factors such as VEGF to activate endothelial cell growth. VEGF isoforms, particularly VEGFA, significantly promote angiogenesis in the wound healing process after cutaneous injury. Thus, we assessed the expression levels of VEGFA, PI3K, and Akt, as well as the phosphorylation status of Akt in wound granulation tissue from all treatment groups at each time point after injury (*n* = 3). Although VEGFA expression levels relative to *β*-actin in the DBLTG-treated group were significantly higher than those in the model control and vehicle control groups, no remarkable differences were observed between the control groups (Figures 5(a) and 5(b)). Moreover, Akt phosphorylation was markedly increased by DBLTG treatment and gradually declined from day 3 to day 14 after surgery; however, expression levels of PI3K and Akt in the DBLTG-treated group were similar to those in both control groups during the entire wound healing process. These results demonstrate that DBLTG activates the VEGF/PI3K/Akt signaling pathway* in vivo*.

Synthesis of the extracellular matrix (ECM), including type I and type III collagen, is another essential orderly sequenced event in wound healing, and it is an important TGF-*β*/Smads signaling target. Therefore, we evaluated the expression levels of TGF-*β*1, Smad2/3, Smad4, and p-Smad2/3 in wound granulation tissue from the different treatment groups at all the time points after surgery (*n* = 3). Expression of TGF-*β*1 relative to *β*-actin in the DBLTG-treated group was remarkably higher than that in both control groups, while the relative expression of TGF-*β*1 in the model control group closely resembled that of the vehicle control group (Figure 5(c)). Furthermore, Smad4 expression and Smad2/3 phosphorylation were notably stimulated by DBLTG administration, whereas there were no significant differences in the relative expression of Smad2/3 between the DBLTG-treated group and both control groups during the postoperative process (Figure 5(d)). These findings show that DBLTG induces the TGF-*β*/Smads signaling pathway* in vivo*.

## 4. Discussion

Wound healing is a complex, dynamic, and systematic process involving the interaction of multiple growth factors, cells, and matrix molecules that can be divided into four phases that overlap in time and space, including a hemostasis phase, inflammatory phase, proliferative phase, and remodeling phase. Previous studies suggest that TCMs and extracts, such as* Panax ginseng*, AS, AR,* Cinnamomum cassia*, and* Salvia miltiorrhiza*, could be used as therapeutic agents for wound repair by stimulating angiogenesis and collagen synthesis [18].

In this study, we prepared DBLTG by loading DBE derived from DBD consisting of AS and AR into liposomes and then dispersing them in an in situ gel, thereby forming a wound dressing. This method resulted in a novel sustained-release drug delivery system for cutaneous wound healing that combines the curative effects of DBE with the advantages of modern preparation technology. We then investigated the therapeutic efficacy of DBLTG to improve dermal wound healing and to activate the VEGF/PI3K/Akt and TGF-*β*/Smads signaling pathways in dorsal full-thickness excisional wounds in rats.

Macroscopic results demonstrate that the wound area in the DBLTG-treated group was significantly smaller than that in both control groups (Figure 1) during the entire wound healing process. These results indicate that contraction is markedly accelerated in DBLTG-dressed wounds in rats. Moreover, histological analysis of wound tissues stained with H&E (Figure 3) showed that DBLTG treatment decreases inflammatory cells, alleviates edema, facilitates granulation tissue formation, stimulates macrophages, increases vascular endothelial and fibroblast proliferation, promotes angiogenesis and collagen synthesis, and improves epithelial regeneration in a rat excisional wound model. These findings agree with the macroscopic observation mentioned above and indicate that DBLTG application accelerates rat full-thickness excisional wound healing.

Macrophages play a vital role in all stages of the wound healing process and can influence the proliferation and differentiation of vascular endothelial cells, fibroblasts, keratinocytes, and other types of cells via growth factor secretion. In the present study, more macrophages were observed in the DBLTG-treated group from 3 to 7 days after surgery than those in the control groups. Recently, astragaloside IV (AS-IV), a major active component extracted from AR, has been shown to improve wound healing in full-thickness skin wounds of streptozotocin-induced diabetic mice, likely through increased activation of macrophages [19]. These data suggest that the beneficial effects of DBLTG on macrophage secretion might be related to AR and its active ingredients.

Fibroblasts are the most common cells in animal connective tissue and play an essential role in wound tissue reconstruction. When skin injuries occur, fibroblasts immediately gather and proliferate in the wound bed and then synthesize and secrete a large amount of collagen fibers and ECM components. Together with newly formed microvessels, the collagen fibers and ECM components form granulation tissue to fill the wound defects and create conditions for epithelial regeneration. Recent studies have shown that the application of AS and AR promotes fibroblast proliferation, resulting in an improvement in wound healing. An ethanol extract of AS along with ferulic acid, its primary active constituent, was shown to reduce the level of intracellular reactive oxygen species (ROS) and promote fibroblast viability and mobility [20]. Furthermore, AR coupled with* Rehmanniae radix* (RR) enhances fibroblast viability and migration* in vitro* and improves diabetic wound healing* in vivo* [21, 22]. In this study, fibroblasts were rapidly growing in an orderly arrangement and formed a cross-linked network in the DBLTG-treated group from 7 to 14 days after injury. Conversely, fewer fibroblasts and more edema and inflammatory cells were found in both control groups. These results demonstrate that the AS and AR components of DBLTG are responsible for the increased aggregation and proliferation of fibroblasts in rat excisional wounds.

The angiogenic effects of AS and AR have been confirmed in previous studies. An AS extract promoted angiogenesis in HUVECs* in vitro* and zebrafish* in vivo*, likely by increasing VEGF expression and inducing JNK 1/2 and p38 phosphorylation [23]. Similarly, an AR aqueous extract exhibited significant angiogenic activity in zebrafish embryos, as determined via evaluation of* VEGFA*,* KDR*, and* Flt-1* gene expression, as well as changes in cell proliferation and the cell cycle in human microvascular endothelial cells [24]. Moreover, AS combined with AR exerted a synergistic effect on HUVEC proliferation by stimulating VEGF expression and increasing the proportion of cells in the S phase [12]. In the present study, newly formed blood vessels were recognized by anti-CD34 antibodies, and the immunohistochemical staining results showed that CD34 expression in granulation tissues from full-thickness excisional wounds in rats at each time point after surgery was markedly higher in the DBLTG-treated group than that in the control groups (Figure 4(a)). The underlying mechanisms might be associated with the migration of AS and AR-activated vascular endothelial cells in conjunction with the activity of adhesion molecules and chemotactic factors, which facilitate vascular endothelium repair and revascularization. In addition, directional assembly of a variety of monocytes, neutrophils, and macrophages induced by inflammatory mediators accelerates angiogenesis directly or indirectly, which is consistent with the observed histological improvements (Figure 3). Along with new blood vessels generation, cell proliferation is also one of the key events needed for new tissues formation during cutaneous wound healing process [25]. We have identified a remarkable increase of cell proliferation in wound granulation tissues from DBLTG-treated wounds at each sampling time as compared to both control wounds (Figure 4(b)).

Collagen is an abundant protein in skin that is used to hold water within the dermis and functions as a matrix to support dermal cells. Among various types of collagen, type I and type III collagen are primarily implicated in the dermal wound healing process, especially during the late stages. We found that secretion of type I and type III collagen is gradually enhanced as rat excisional wound healing progresses, an effect that is remarkably increased by DBLTG administration (Figures 4(c) and 4(d)). These data suggest that DBLTG stimulates type I and type III collagen synthesis to promote dermal wound healing. Moreover, hydroxyproline is a characteristic component of collagen, and its content in wound granulation tissue reflects the degree of epidermal regeneration in the late phase of wound healing. We showed that hydroxyproline content in dermal wounds upon DBLTG treatment was significantly higher on day 14 after injury than that observed in wounds receiving the control treatment (Figure 2). This finding also implies that DBLTG-enhanced cutaneous wound healing is achieved by increasing the secretion of hydroxyproline.

VEGF, a highly specific cytokine for vascular endothelial cells, plays an essential role in angiogenesis, particularly in its initial stages [26]. When VEGF binds to VEGF receptors, it activates several downstream signaling pathways, including the PI3K/Akt, IP3/Ca2+/endothelial nitric oxide synthase (eNOS)/NO, FAK, p38 mitogen-activated protein kinase (MAPK), and Ras/Raf-MEK/Erk pathways [27]. Previous studies have shown that QCSP3/PEGS-FA1.5, a self-healing hydrogel wound dressing, significantly enhances the* in vivo* wound healing process in a full-thickness skin wound model by stimulating the gene expression of growth factors, including* VEGF* [28]. Further, an N-butyl alcohol extract from* Hibiscus rosa-sinensis* L. flowers improves wound healing by enhancing macrophage activity and accelerating angiogenesis and collagen synthesis mediated by VEGF and TGF-*β*1, as evidenced by faster wound contraction and greater epidermal epithelialization in rat excisional skin defects [29]. Moreover, Lee et al. [30] revealed that PI3K/Akt signaling is essential for the epithelial-mesenchymal transition (EMT) and keratinocyte migration that, when dysfunctional, leads to compromised wound healing. Further, Yang et al. [31] proved that PI3K/Akt signaling is indispensable for lucidone-induced cell proliferation and migration of HaCaT cells. Here, we show that DBLTG upregulates the expression of* VEGFA* and the phosphorylation of Akt, suggesting that DBLTG-induced improvements in angiogenesis in rat dorsal full-thickness excisional wounds are attributable to the activation of VEGF and its downstream signal transducers, as confirmed by histological and immunohistochemical analysis.

TGF-*β*1 is a crucial cytokine that stimulates cell proliferation and regulates ECM synthesis [32]. When bound to TGF-*β*1, TGF-*β* type II receptor directly recruits and phosphorylates TGF-*β* type I receptor. Once recruited to the activated TGF-*β* receptor complex, Smad2/3 is phosphorylated and integrates with Smad4 to generate a heterodimeric complex. Subsequently, the Smad complex translocates from the cytoplasm to the nucleus and initiates the expression of downstream target genes [33]. Previous research [34] reported that* Eleutherine indica* L. methanolic extract (2.5%,* w/w*) accelerates cutaneous wound healing in a circular excision model of male Wistar rats by stimulating Smads-mediated collagen production. Zhang et al. [35] found that NF3, a Chinese herbal prescription consisting of AR and RR at a ratio of 2 : 1* (w/w)*, enhances Hs27 human skin fibroblast cell migration and upregulates the expression of genes involved in ECM deposition, including* COL1A1* and* COL3A1*, by activating the TGF-*β*/Smads pathway. These results reveal that improvements in collagen production during the wound healing process are mediated by the TGF-*β*/Smads pathway. Our study also demonstrates that DBLTG promotes the expression of TGF-*β*1 and phosphorylation of Smad2/3, implying that DBLTG-enhanced COL1*α*1 and COL3*α*1 synthesis in wound models is likely attributable to activation of the TGF-*β*/Smads signaling pathway, as shown by immunohistochemical analysis.

## 5. Conclusion

In the present study, DBLTG was successfully prepared by loading DBE into liposomes and then dispersing them in a thermosensitive gel. DBLTG showed greater therapeutic efficacy, as evidenced by faster wound closure; histological improvements; higher hydroxyproline levels; and increased CD34, Ki67, Col1*α*1, and Col3*α*1 expression levels in wound granulation tissues compared with those of the control groups* in vivo*. Furthermore, we demonstrate that DBLTG activates the VEGF/PI3K/Akt and TGF-*β*/Smads signaling pathways, which might contribute to its ability to accelerate full-thickness excisional wound healing in rats. In summary, DBLTG is a promising wound dressing for dermal wound healing. Further, loading Chinese medicine formula extracts into topical sustained-release drug delivery systems is a novel and beneficial strategy for improving cutaneous wound healing.
